# Delayed transfer of care from NHS secondary care to primary care in England: its determinants, effect on hospital bed days, prevalence of acute medical conditions and deaths during delay, in older adults aged 65 years and over

**DOI:** 10.1186/1471-2318-9-4

**Published:** 2009-01-22

**Authors:** Krishantha H Jasinarachchi, Ibrahim R Ibrahim, Breffni C Keegan, Rajaratnam Mathialagan, John C McGourty, James RN Phillips, Phyo K Myint

**Affiliations:** 1 Norfolk and Norwich University Hospital, Colney Lane, Norwich, NR4 7UY, Norfolk, UK; 2 Erne Hospital, Enniskillen, County Fermanagh, BT74 6AY, UK; 3 Queen Elizabeth Hospital, Gayton Road, King's Lynn, PE30 4ET, Norfolk, UK; 4 School of Medicine, Health Policy and Practice, University of East Anglia, Norwich, NR4 7TJ, Norfolk, UK; 5 Clinical Gerontology Unit, Department of Public Health and Primary Care, Addenbrooke's Hospital, Cambridge, CB2 0QQ, UK

## Abstract

**Background:**

The delay in discharge or transfer of care back to the community following an acute admission to the hospital in older adults has long been a recognized challenge in the UK. We examined the determinants and outcomes of delayed transfer of care in older adults.

**Methods:**

A prospective observational study was conducted in a district general hospital with a catchment population of 250,000 in England, UK. Those >= 65 years admitted to two care of the elderly wards during February 2007 were identified and prospectively followed-up till their discharge. Data was presented descriptively.

**Results:**

36.7% (58/158) of patients had a delay in transfer of care. They tended to be older, had poorer pre-morbid mobility, and were more likely to be confused at the time of admission. Compared to the 2003 National Audit Report, a significantly higher percentage (29.3%vs.17%) awaited therapist assessments or (27.6%vs.9%) domiciliary care, with a lower percentage (< 1%vs.14%) awaiting further NHS care. Of 18 in-patient deaths, five occurred during the delay. Seven patients developed medical conditions during the delay making them unfit for discharge. The number of extra bed days attributable to delayed discharges in this study was 682 (mean = 4.8) days.

**Conclusion:**

Awaiting therapy and domiciliary care input were significant contributing factors in delayed transfer of care. Similar local assessments could provide valuable information in identifying areas for improvement. Based on available current evidence, efficacy driven changes to the organisation and provision of support, for example rapid response delayed discharge services at the time of "fit to discharge" may help to improve the situation.

## Background

The delay in discharge or transfer of care back to the community following an acute admission to the hospital in older adults has long been a recognized challenge in the UK [1-3]. This has implications both for the patients' well being and cost to the NHS organisations [4]. Given the importance of this issue, the UK Government undertakes continual interventions such as increased funding [5] or introducing new legislation [6] in attempts to improve the situation.

The Department of Health has defined a delayed transfer of care as "Occurring when a patient is ready for transfer from a general and acute hospital bed, but is still occupying such a bed. A patient is ready for transfer when: (1) a clinical decision has been made that the patient is ready for transfer; (2) a multidisciplinary team decision has been made that the patient is ready for transfer; and (3) the patient is safe to discharge/transfer" [7].

The National Audit Office reported its findings in 2003 on the common contributory factors for delayed transfer of care across the UK [8]. While there is increasing pressure on clinical teams, the NHS organisations, Primary Care Trusts and Social Services, the problem of delayed discharge is compounded by the lack of community beds due to recent closure of nursing homes and community hospitals in England. Glasby and colleagues [9] reviewed 21 studies examining the delayed transfer of care and found that there were different rates and causes of delayed discharges in different settings. However, there is limited information on the effect of delay on patients in terms of prevalence of further acute illnesses and in-patient mortality during the delayed period. There is also lack of data on how delay itself and the further delay due to subsequent illness episodes would manifest on a clinically meaningful outcome of length of stay or extra bed days.

The main objectives of the current study were (1) to explore the factors that are related to delayed transfer of care from an acute hospital to the community after a patient has been declared medically fit to transfer; (2) to determine the effect of delayed transfer of care on health services in terms of extra acute hospital bed days; and (3) to examine the patient related outcomes in terms of prevalence of illness episodes and deaths during the delayed period in an older hospitalised population. We also examined the factors related to delayed transfer of care compared to national results benchmarked by the National audit data published in 2003.

## Method

We conducted a prospective observational study in a UK district general hospital with a catchment population of approximately 250,000. Our local hospital covers the town of King's Lynn and surrounding rural areas in West Norfolk, England, UK. Patients on two care of the elderly wards who were present as inpatients at the time of study commencement (1^st ^February 2007) and every patient admitted over one calendar month (i.e. those who were admitted till end February 2007) were included in the study. Each patient was followed up till their discharge, either alive (discharge/transfer back to the community) or dead (in-patient death). All patients aged 65 years and over were included in this current study with the exception of those whose index hospital admission was due to terminal illness which was expected to result in death during their admission. This project was conducted as a baseline assessment of delayed transfer of care in the Queen Elizabeth Hospital as a clinical governance project. We present the data in anonymised and aggregated fashion. Therefore, LREC approval was not sought.

Identification of delayed transfer of care was carried out using the official definition of the Department of Health [7]. For this study purpose, patients who remained as in-patients for in-patient rehabilitation, and those who were discharged within 24 hours of being declared medically fit were not regarded as cases of delayed transfer of care. Data collection was carried out prospectively from admission data and medical records using a standardised data collection sheet. Case notes were reviewed by the two investigators (KHJ and IRI). Consultant physicians (BCK, RM, JCM and JRNP) who looked after the patients during the study period identified those patients who were medically fit. Similar categories of delayed transfer of care were used as in the 2003 National Audit report for direct comparison [8]. The patients who had delayed transfer of care were followed for any illness occurring during their prolonged hospital stay including death.

Data were analysed using SPSS for Windows Version 14.0 (SPSS Inc, Chicago, Il, USA). Data were presented descriptively. The reasons for delayed transfer of care in this series were compared to the 2003 National Audit Data. Using logistic regression, the likelihood of being in the delayed discharge category was assessed for patient related factors such as age, sex, clinical parameters at the time of admission including systolic blood pressure, pulse rate, albumin level, urea level, creatinine level, C-reactive protein (CRP) level and their effort tolerance.

## Results

A total of 158 patients were eligible to be included in the study. There were 79 males and 79 females (50% each). Their ages ranged from 66 to 98 years (median = 82.5 years). The majority of patients were free living that is independently living in their own residence: 121 (76.6%) from their own homes; 21 (13.3%) from a residential home; 9 (5.7%) from a nursing home; and 5 (3.2%) from sheltered accommodation. One was admitted from an older mentally infirm unit and another from a psychiatric unit.

The number of co-morbidities ranged from 0–9 with 60% of older people having at least three co-morbid conditions. In terms of mobility, 63 (41.4%) used no walking aids and were able to walk greater than 50 metres without requiring rest. 15 (~10%) were heavily dependent (hoisted, bed bound or could only bear weight). The number of usual medications ranged from 0–15. The majority (131/156, 84%) received four or more regular medications.

There were a total of 58 delayed discharges observed in this study population. Comparison of sample characteristics between those whose discharge was delayed and those whose discharge was timely is presented in table 1. Those in the delayed discharge group were significantly older, had lower levels of mobility, and were more likely to be confused. Interestingly, those who had delayed discharges as a group had lower C-reactive protein (CRP) levels at admission compared to those who had timely discharges. Reasons for delays are presented in the table 2 in comparison to the 2003 National Audit data. A significantly higher percentage (29.3% vs. 17%) in this series was awaiting further therapy assessments pre-discharge. A lower percentage (< 1% vs. 14%) awaited further NHS care, but a higher percentage (27.6% vs. 9%) awaited domiciliary care to be in place. The average extra length of stay due to delayed discharges was 4.84 days with a maximum delay of up to 150 days. Total number of extra bed days from 58 delayed discharges in this cohort was 682 bed days.

**Table 1:** Comparison of sample characteristics

	**No delay**	**Delay**	**p**
Age (yrs)	81.3 (7.4)	84.4 (7.2)	0.012*
Sex			0.73
Male	43 (51.2)	28 (48.3)	
Female	41 (48.8)	30 (51.7)	
Residence			0.36
Home	65 (77.4)	42 (72.4)	
Residential home	13 (15.5)	7 (12.1)	
Nursing home	3 (3.6)	6 (10.3)	
Sheltered accommodation	2 (2.4)	3 (5.2)	
Psychiatric unit	1 (1.2)	0	
			
Mobility			0.05*
Independent (>= 50 yards)	40 (50.6)	17 (29.8)	
Independent (< 50 yards)	7 (8.9)	15 (26.3)	
Walk with stick	13 (16.5)	5 (8.8)	
Uses walking frame	13 (16.5)	12 (21.1)	
Uses wheel chair	1 (0.7)	2 (1.5)	
Hoisted	2 (2.5)	2 (3.5)	
Bed bound	3 (3.8)	3 (5.3)	
Weight bear/transfer only	0	1 (0.7)	
			
Symptom duration (days)	6.5 (20)	8.3 (25)	0.64
Number of co-morbidities	3.0 (1.5)	2.8 (1.7)	0.64
Effort tolerance (metres)	113 (304)	39 (52)	0.18
Presence of confusion	9 (10.7)	18 (31.0)	0.002*
Systolic BP (mmHg)	134 (31)	129 (29)	0.34
Pulse rate (beats/min)	80 (16)	81 (18)	0.84
C-reactive protein	82 (84)	52 (64)	0.026*
Urea (mmol/L)	10.9 (7.2)	9.3 (5.6)	0.16
Creatinine	139 (76)	131 (77)	0.53
Albumin	31.0 (5.3)	32.3 (4.4)	0.16
Number of medications	6.5 (3.7)	7.4 (3.8)	0.14

Comparison of baseline characteristics between those whose discharges were delayed and those who did not in 158 elderly inpatients. Values presented are means (sd) for continuous data and number (%) for categorical data. * = statistically significant differences in sample characteristics between two groups

**Table 2:** Causes of delayed discharges

	**National Audit (2003)**	**Current study (2007)**
	**Percent**	**Number (%)**
Awaiting residential/nursing home place	26	15 (25.9)
Awaiting assessment of needs	17	17 (29.3)
Awaiting further NHS care	14	1 (0.6)
Awaiting placement of patient's choice	10	-
Awaiting public funding	13	-
Awaiting domiciliary care	9	16 (27.6)
Others*	-	9 (15.5)

Causes of delayed discharges in the current study in comparison to the National Audit Data (2003). * = causes for other reasons include waiting coronary angiogram date (n = 1), awaiting family transport (n = 1), awaiting a rehabilitation bed (n = 2), awaiting hospital transport (n = 2), awaiting case conference (n = 1), awaiting podiatrist (n = 1), family reason (n = 1).

There were 18 in-patient deaths in this cohort. The primary causes of deaths were two due to carcinoma of lung, seven from bronchopneumonia, two from acute renal failure, one each from metastatic cancer, congestive cardiac failure (CCF), cholecystitis, acute coronary syndrome, pulmonary embolism (PE), respiratory failure and *Clostridium difficile *diarrhoea. Of these, five deaths occurred after initially being declared medically fit. These included one death each from bronchopneumonia, CCF, cholecystitis, PE and *Clostridium difficile *diarrhoea.

Out of 58 cases who had delayed discharges, seven developed at least one medical condition making them unfit again for discharge and subject to further delay. These included urinary tract infection (UTI), recurrent dizziness, leg swelling, poor oral intake, lower respiratory tract infection (LRTI), bronchopneumonia and *Clostridium difficile *diarrhoea. One of them developed further UTI and later on developed LRTI.

The likelihood of being in the delayed discharge category was not associated with factors such as sex (Odds Ratio 1.12; 95% confidence interval 0.58, 2.20), systolic blood pressure at the time of admission (1.00; 0.99, 1.02), pulse rate (1.00; 0.98, 1.02), albumin level at the time of admission (0.95; 0.88, 1.02), urea level (1.04; 0.98,1.10), creatinine level (1.00; 1.00, 1.02), or effort tolerance (1.01; 1.00,1.02) but appeared to be associated with advancing age (0.94; 0.90, 0.99, p = 0.014) and marginally associated with CRP level (1.01; 1.00, 1.01, p = 0.032).

## Discussion

In this study we examined the patient related factors which were associated with delayed discharges or transfer of care, and examined their impact on patients and the NHS in terms of extra bed days wasted. Our study confirmed that delay in transfer of care was associated with substantial additional hospital stay, occurrence of acute illness episodes and death during the delay.

Falcone and colleagues performed a study exploring the determinants of delayed discharges of older hospital patients over a quarter of century ago in a US setting [10]. They found that some policy-relevant patient characteristics such as requirement for heavy care, race, source of reimbursement, and whether or not there was a financial problem in arranging discharge were associated with delay. In their study, hospital features such as bed capacity, occupancy rate, and total revenues were also correlated with delay. They concluded that the delay problem warrants more intensive analysis, particularly regarding financial problems encountered at discharge, and race. They also thought there to be a need to recognize the increasing preponderance of a new type of heavy care patient via more appropriate reimbursement levels and "transitional care" services.

We found that older age, poorer pre-morbid mobility and presence of confusion at the time of admission were significantly higher in the delayed group. Similar to our results, Carter and colleagues [11] reported that neurological disability and cognitive impairment with unsuitable accommodation were associated with delayed discharges in relatively younger patient group (18–70 yrs) in Oxfordshire acute hospitals. The Information and Statistics Division (ISD) NHS Scotland reported that probability of experiencing a delay in discharge is strongly related to age, physical and mental frailty and weakly related to number of previous emergency admissions [12]. Glasby and colleagues [9] previously reported that there were different rates and causes of delayed discharges in different settings. Therefore, it is not surprising to observe that our local data is different from the National audit data in some categories of delay.

Interestingly, lower CRP level is associated with borderline significance of being delayed in logistic regression models. This may be due to the fact that CRP usually lags behind the symptom onset; it was not measured in all cases and hence the lack of statistical power. The result may also be explained by the fact that those with raised CRP levels had clearcut medical crisis (i.e. infection) rather than multi-factorial functional decline whose discharges were likely to be delayed.

Almost a third of the patients in this series waited on domiciliary care arrangement before discharge (27.6%) which was a 3 times higher incidence than national figures. This may be due to differences in funding stream between different areas of the country. We observed similar figures for delays relating to the availability of nursing home and residential home places to those recorded in the national audit. This probably indicates that there is little evidence of improvement in addressing the problem of shortages of care homes in the UK. As rehabilitation includes both assessment and resettlement, the apparent bed day loss in the current study may be due to the fact that both processes have been slower than ideal.

Weissert and Cready [13] reported that-where nursing home beds are in short supply- other factors are more important in determining delayed transfer in care. The authors stated that if the study findings were replicated in other areas with perceived nursing home bed shortages, there appeared to be important implications not only for the usefulness of nursing home case-mix reimbursement and subacute levels of nursing home care, but for nursing home bed-need estimates too, as well as for Medicaid eligibility, determination practices and civil rights law enforcement.

There have been a number of recent key policies and initiatives by the Department of Health in England to achieve more effective discharge. These included (1) the development of a wide range of intermediate care services with over £500 million in funding, (2) an additional Delayed Discharges Grant of £100 million per annum to encourage local authorities to work with health partners to tackle the causes of delay within their systems, (3) the creation in January 2002 of the ...Health and Social Care Change Agent Team to support the implementation of the NSF for Older People, by developing a single system of health and social care and to work with local agencies to reduce delayed hospital discharges, (4) the publication of an updated ...Hospital Discharge Workbook in 2003 emphasizing the need for a whole systems approach, and (5) The implementation of the Community Care (Delayed Discharges etc.) Act 2003, to impose financial penalties on social services departments unable to arrange social care packages for patients medically fit for discharge within 3 days widely known as reimbursement [14].

Since the 2003 legislation, there has been some evidence of growth in 'step-down' intermediate care and rehabilitation facilities but with older people experiencing multiple moves post discharge. There are indications that some staff find the pressure for a quick discharge unhelpful regarding the quality of service they can offer, particularly influencing the input of social workers advising older people on post discharge options for care. There also appeared less choice and involvement for older patients needing to make life changing decisions speedily, and some evidence of an increase in readmissions to hospital [14].

Lees et al [15] also recently cautioned that delayed discharge fines increase pressure on trusts to prevent delays. Hudson and Herbert [16] also highlighted the significant potential for conflict between organisations and stated that there was no benefit for service users based on a simulation exercise of charging for delayed discharges. In their study, participants thought the charging system would work against the development of community services. They favoured joint NHS/local authority approaches to the development of an effective system for preventing delayed discharges. The delayed discharge problem is not exclusive to UK; it was a problem in other western countries [17-20]. There are also anecdotal data suggesting that this phenomenon has been observed in all settings where hospital financing changed from cost based to price based.

Victor et al [21] found that considerable delay in discharging older people from hospital originated from administrative/organisational issues which are compounded by social services resource constraints. There is evidence of positive effect of organisational changes driven by the government's reimbursement scheme, for example the provision of 'delayed discharge teams' that offer services free for up to 6 weeks post discharge. These teams address the fact that main stream services are slow to respond to rapid discharge decisions. A recent study by Baum and colleagues [22] found that there are ranges of factors that reportedly contribute to low rate of delays.

Naturally there are limitations in our study. Although medically fit status for discharge was decided by consultant, data were collected by two junior doctors and inter-observer agreement was not tested. Therefore, there may be some misclassification of category of delayed transfer accounting for some discrepancies from the National audit data. However, the point at which a patient is "medically fit for discharge" is a subjective judgement which would have been implicit in the national audit. Nonetheless, the data collection involved discussion between data collectors as well as prior agreement with regards to categorisation of delayed transfer. Moreover, categorisation was done in consultation with senior colleagues (specialist registrars or consultants) and other multi-disciplinary team members including nursing staff, physiotherapists, occupational therapists and social workers. There may have been other differences which affect the comparison.

We defined delayed discharges as discharges delayed > 24 hours after being declared medically fit. This can lead to an underestimation of the effect of delay on total number of bed days wasted. The results may be driven by the time of the year, and because the study was performed in the middle of the winter period in the UK, we might have overestimated the impact of delayed transfers. We did not follow up those who were regarded as timely discharges. Therefore, we are not able to conclude firmly that delayed discharges were associated with higher than expected morbidity and mortality. Future studies, however, should be designed to address this. Nevertheless, there is evidence that people, especially older people in hospital beds, are at risk from Hospital Acquired Infections (HAIs), malnutrition, depression and falls. They also fail to receive continuing rehabilitative therapy which leads to dependency. These factors are pertinent to the types of patient described in this report and some of deaths due to conditions such as *Clostridium difficile *diarrhoea, PE and bronchopneumonia which occurred during the delay.

Another limitation of the study is that while there is an advantage to focus on one hospital serving one catchment area, the disadvantage is that part of the reason for the back up of older patients in hospital beds is probably due to local factors, for example the lack of community based resources such as nursing home beds or home care services. Having information on these factors and comparing between more than one area would allow the introduction of these factors into the model. Finally, we do not have data on how much help people had at home normally prior to hospital admission. As the services may be difficult to restart after a period of hospitalisation, this may have contributed in a delayed discharge. Therefore there is potential for improvement if the hospital and social services are co-ordinated in the way that such wait can be minimised. The studies specifically looking at this dimension of potential contributor of delayed discharges may help to improve services in the future. We were not able to take into account the presence or absence of and the capacity of the formal and informal (family) social support. The fact that there were little associations between the physiological factors and being delayed discharge may be as the result of inadequate adjustments due to lack of social data or due to other unknown confounders.

It is possible that the reported situation could be an underestimate of the actual magnitude of the problem. If our results were extrapolated to approximately 200 NHS Trusts in England, the impact of delayed transfer of care on morbidity, mortality and financial implication to the NHS could be substantial. The cost spent for associated bed days lost, morbidity and mortality could have been invested to improve social services, therapist input and care home beds.

## Conclusion

In conclusion, although our results may not be applicable to all NHS Trusts, this study highlights the fact that urgent and more detailed attention is required to address delayed transfer of care in the UK and beyond if we are to provide delivery of care and health services more efficiently in the future.

## Competing interests

The authors declare that they have no competing interests.

## Authors' contributions

KHJ developed the idea. KHJ and PKM designed the project. KHJ and IRI collected the data. All authors had involvement with some of the patients' care included in the study. KHJ and PKM prepared the draft manuscript. All authors contributed in the writing of the paper. All authors read and approved the final manuscript.

## Pre-publication history

The pre-publication history for this paper can be accessed here:


